# Thickening of T_1_ Precipitates during Aging of a High Purity Al–4Cu–1Li–0.25Mn Alloy

**DOI:** 10.3390/ma12010030

**Published:** 2018-12-21

**Authors:** Ines Häusler, Reza Darvishi Kamachali, Walid Hetaba, Birgit Skrotzki

**Affiliations:** 1 Federal Institute for Materials Research and Testing (BAM), Department 5: Materials Engineering, 12205 Berlin, Germany; haeusler@tu-berlin.de; 2 Institute of Optics and Atomic Physics, Technical University of Berlin, 10623 Berlin, Germany; 3 Max-Planck-Institut für Eisenforschung GmbH, Max-Planck-Straße 1, 40237 Düsseldorf, Germany; kamachali@mpie.de; 4 Department of Inorganic Chemistry, Fritz-Haber-Institute of the Max-Planck-Gesellschaft, 14195 Berlin, Germany; hetaba@fhi-berlin.mpg.de; 5 Department of Heterogeneous Reactions, Max-Planck-Institute for Chemical Energy Conversion, 45470 Mülheim an der Ruhr, Germany

**Keywords:** Al-Cu-Li-alloy, precipitation, T_1_ precipitate, microstructure evolution, thickening, creep, volume fraction, number density, strain difference

## Abstract

The age hardening response of a high-purity Al–4Cu–1Li–0.25Mn alloy (wt. %) during isothermal aging without and with an applied external load was investigated. Plate shaped nanometer size T_1_ (Al_2_CuLi) and θ′ (Al_2_Cu) hardening phases were formed. The precipitates were analyzed with respect to the development of their structure, size, number density, volume fraction and associated transformation strains by conducting transmission electron microscopy (TEM) and scanning transmission electron microscopy (STEM) studies in combination with geometrical phase analysis (GPA). Special attention was paid to the thickening of T_1_ phase. Two elementary types of single-layer T_1_ precipitate, one with a Li-rich (Type 1) and another with an Al-rich (Defect Type 1) central layer, were identified. The results show that the Defect Type 1 structure can act as a precursor for the Type 1 structure. The thickening of T_1_ precipitates occurs by alternative stacking of these two elementary structures. The thickening mechanism was analyzed based on the magnitude of strain associated with the precipitation transformation normal to its habit plane. Long-term aging and aging under load resulted in thicker and structurally defected T_1_ precipitates. Several types of defected precipitates were characterized and discussed. For θ′ precipitates, a ledge mechanism of thickening was observed. Compared to the normal aging, an external load applied to the peak aged state leads to small variations in the average sizes and volume fractions of the precipitates.

## 1. Introduction

Al–Li base alloys represent an attractive material class for weight critical applications in aviation and space flight industry. These includes, e.g., fuselage and upper wing structures or cryogenic tanks [1]. These alloys are mainly strengthened by precipitation hardening. In the Al–Cu–Li system, δ′ (Al_3_Li), θ′ (Al_2_Cu) and T_1_ (Al_2_CuLi) are the main phases that contribute to the precipitation hardening [2,3,4,5,6,7,8]. The presence and amount of these phases strongly depend on the heat treatment and Cu/Li ratio in the alloy composition [2,9]. While the precipitation of δ′ and θ′ phases have been comprehensively investigated, the mechanism of formation and growth of the T_1_ phase is still under debate. In some early studies, Nobel and Thompson [2] and Cassada et al. [10,11] proposed that the nucleation, growth and thickening of T_1_ precipitate occur by dissociation of dislocations and propagation of ledge kinks. This mechanism assumes T_1_ as a four-layer platelet structure in accordance with Howe et al. [12] who proposed a structure for T_1_ phase correcting an earlier structure suggested by Huang and Ardell [13]. In the light of advanced electron microscopy, however, a more complex structure for the T_1_ phase has been observed that also motivates possible revision of previous theories of growth for the T_1_ phase: Recent studies on the single-layer T_1_ precipitate could be best explained by a model proposed by van Smaalen [14], while the earlier models by Huang and Ardell [13] and Howe et al. [12], based on which the growth mechanism for T_1_ precipitate is described, are completely ruled out. This was first discussed by Donnadieu et al. [15] using scanning transmission electron microscopy (STEM) and small angle X-ray scattering (SAXS) studies, and later confirmed independently by Dwyer et al. [16] using STEM and density functional theory (DFT) studies. The characteristic feature of van Smaalen’s model is the corrugated arrangement of the atomic layers parallel to the precipitate plane which suggest large rearrangement of atoms corresponding to the {111} parent planes in the Al Matrix. Very recently, Kim et al. [17] performed DFT calculations and proposed a modified version of the van Smaalen structure, in which the arrangement of Li atoms in the interface layer are different from all previous models. Using a cluster expansion approach, they also found the possible stable T_1_ phase. The calculations, however, are at 0 K and still do not fit in the phase diagram of the ternary Al–Cu–Li system.

The thickening of the T_1_ precipitates seems to be an even more complex problem: It is typically observed that the T_1_ phase first forms as a thin platelet precipitate (single-layer) quite stable in its thickness while it grows normally along its edge [10]. The thickness of a single-layer T_1_ precipitate is found to be less than 1 nm differing for different alloy compositions [18]. At elevated temperatures or very long aging times, an abrupt thickening of T_1_ precipitate has been observed, seemingly by integer multiplication of the initial T_1_ single-layers accompanied by strong composition variations normal to the precipitate plate [10,11,19]. The details of this thickening were not investigated in detail in the previous studies. In particular, the structure and composition of the precipitate/matrix interface, on which the thickening occurs, is still unclear. Although it was found that, in agreement to the van Smaalen’s structure, an Al–Li layer bridges the precipitate to the Al matrix phase [16], the interface layer is also evidenced to be distorted, being closer to the atomic structure of the Al matrix [15]. Hence, it is not known whether this layer belongs to the precipitate or to the Al matrix phase. In addition, segregation of secondary solute elements such as Ag has been observed [20,21], which suggests a higher energy state of the precipitate–matrix interface.

The effects of transformation strains and external stresses on the evolution of precipitates in Al alloys are widely known [22,23,24,25,26,27,28,29,30]. For the T_1_ precipitate, it is known that dislocations may play a role in both nucleation and growth. Cassada et al. [11] showed that stretching the solutionized material prior to aging at 190 °C can largely assist with the nucleation and formation of T_1_ precipitates and argued that dislocation formation and dissociation play a critical role in its thickening. Atom Probe Tomography (APT) studies revealed that segregation of Mg and Cu at the dislocations correlates with nucleation of T_1_ precipitates [21]. However, formation of the T_1_ phase without predeformation has also been reported [5]. Other structural defects can also play a role in the T_1_ precipitation process [31]. A defected T_1_ structure can form due to the interaction with dislocations. It is found that T_1_ precipitates can be sheared by dislocations even when they reach a thickness of several nanometers [32], which improves the formability of the alloy. The stable and small thickness of the T_1_ precipitates indicates a large elastic anisotropy of this phase, but the strain associated to its formation is not yet fully uncovered due to the unknown phase structure.

In this study, comprehensive transmission electron microscopy (TEM), high resolution scanning transmission electron microscopy (HR-STEM) and geometrical phase analysis (GPA) measurements were conducted to gain insights regarding the evolution of T_1_ and θ′ precipitates. Special attention was paid to the formation and thickening of the T_1_ phase. We considered a rather high-purity model alloy (Al–4Cu–1Li–0.25Mn alloy (wt. %)) that is similar to the technical alloy AA2195 with respect to Cu, Li and Mn, but does not contain secondary alloying elements. The high purity allowed better characterization of the precipitation mechanisms without the accelerating effects due to additional alloying elements [20,21]. In this alloy, both T_1_ and θ′ may form and grow. The details of peak hardening response of the current alloy have been previously studied [33]. In the current study, we extended the previous work by investigating long term aging and aging under external load (creep conditions). In particular, the layering sequence of T_1_ phase and the role of the interfacial layer during its thickening were discussed. The strains normal to the T_1_ precipitate plane were measured along the thickening of the precipitate. The effect of external load on the evolution of these precipitates were studies as well. A mechanism of thickening was proposed for the T_1_ phase that is associated with the transformation strain accompanying the process. Different defected structures of T_1_ phase were identified and analyzed as well. Furthermore, the size evolution of both T_1_ and θ′ precipitates was studied in detail.

## 2. Materials and Methods

### 2.1. Material and Sample Preparation

The investigated aluminum alloy has a nominal chemical composition of 4% Cu, 1% Li and 0.25% Mn (wt. %; balance Al). The amount of impurities is less than 0.06% [33] (i.e., 0.012 Si, <0.006 Fe, 0.007 Mg, <0.001 Ni, <0.009 Zn, 0.003 Co, 0.004 Sn, 0.005 Bi, 0.0031 Cd, and all other elements < 0.001; all in wt. %). High-purity elements were molten in a vacuum induction furnace and poured into a water-cooled crucible under argon atmosphere. The cast was homogenized at 515 °C for 24 h, water quenched and subsequently extruded at 420 °C (profile cross-section: 15 mm × 70 mm). The final heat treatment process was solutionizing of the profile at 505 °C for 70 min, followed by water quenching. At last, the extruded profiles were stretched by about 2.9% to straighten them. The resulting material was received as semi-finished block profiles with dimensions of 15 mm × 70 mm × 655–785 mm. This material state is designated as “initial state” in the following.

**Figure 1:**
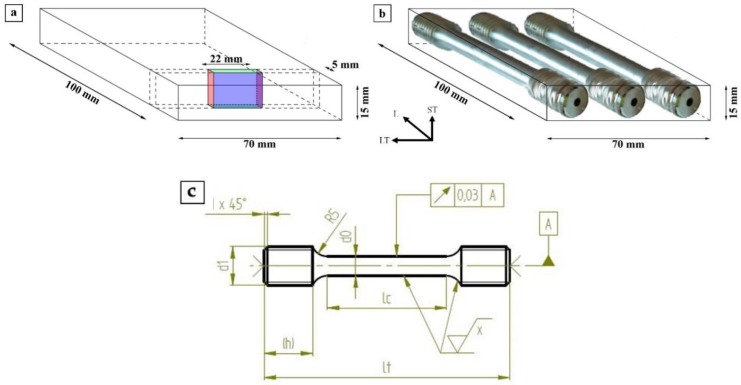
Sampling of specimens from the extruded profile (L, longitudinal direction; LT, long transverse direction; ST, short transverse direction): (**a**) coupons for aging treatments; (**b**) creep specimens*;* and (**c**) creep specimen geometry with dimensions of d0 = 8 mm, d1 = M12, lc = 60 mm, lt = 100 mm, and (h) = 15 mm. Surface finish: R_z_ = 3.2.

The strand was cut into 100 mm long sections, which were afterwards thermally aged to the peak aged (PA) state. The aging was carried out at 180 °C for 17 h in a radiation furnace (ATS model 3710, Applied Test System, Inc. (ATS; Butler, PA, USA); maximum temperature deviation: ±5 °C) according to parameters defined in [33]. Subsequently, coupons and creep specimens were machined from the section, as shown in Figure 1a,b. The dimensions of the coupons, which were used for hardness measurements and serve as isothermally and load-free aged reference samples during the study (called reference sample in the following), were 22 mm × 15 mm × 5 mm. The longitudinal axis of the creep specimens corresponds to the L-axis of the profile. The geometry of the creep specimens is given in Figure 1c. The gauge diameter was 8 mm, while the gauge length was 50 mm.

Table 1 summarizes the conditions (and sample designations) investigated in this paper. *Ref. 0* represents the peak aged (PA) state. The remaining samples received a further isothermal aging treatment after the PA treatment: samples *Ref. 1* and *Ref. 2* were aged at 180 °C for 257 h and 1002 h, respectively. Samples *Creep test 1* and *Creep test 2* were aged under an external load of 155 MPa at the same temperature and for the same time as *Ref. 1* and *Ref. 2*. Aging under load was realized in a standard creep testing rig (constant load lever arm machine, 20 kN capacity). The length change Δ*l* of the specimens was recorded using an extensometer system and then converted into strain (*ε* = Δ*l*/*l*_0_).

The hardness was measured for both the coupons and the creep specimens on the ST-LT plane. The surface was ground with SiC abrasive paper (grit size P600, P1200, P2400) and subsequently polished with diamond suspension (particle size 3 μm and 1 μm). Brinell hardness was determined following DIN EN ISO 6506-1 [34] using hardness tester M4C 025 G3 (EMCO-TEST, Kuchl, Austria). The testing parameters were: ball diameter 2.5 mm, test force 612.9 N, and exposure time between 10 s and 15 s.

For the transmission electron microscopy (TEM) investigation of the creep specimens, samples were cut from the center of the gauge section parallel to the external loading direction (i.e., in the L-LT-plane). Samples of the reference states were taken from the coupons in L-LT-plane. After sawing, all TEM specimens were prepared by conventional technique (grinding and polishing of both sides down to 100 µm, and punching of discs with 3 mm diameter). Finally, the discs were electro-polished using a solution of methanol (CH_3_OH) and nitric acid (HNO_3_) at a ratio of 2:1 at −23 °C. 

High-angle annular dark-field (HAADF) scanning TEM (STEM) images were recorded using the STEM/TEM microscope 2200FS (JEOL, Tokyo, Japan) (acceleration voltage: 200 keV; equipped with an in-column-energy filter and a HAADF detector) at the Federal Institute for Materials Research and Testing (BAM). After recording the HAADF-STEM images, a thickness map of the same area was taken. For this purpose, the area was divided into a grid of 20 × 20 points. The recording and analysis of the low loss region of the electron energy loss spectrum (EELS) of each point of the grid enables the determination of the TEM foil thickness within the grid in terms of units of “mean-free path length” of aluminum [35]. Both convergent beam electron diffraction (CBED) patterns and EEL spectra at the same position were recorded and analyzed for conversion from “mean-free path length” into “nm” [35,36]. The evaluation revealed a mean-free path length of 137 nm [33]. The high-resolution HAADF-STEM images were recorded at the Fritz Haber Institute of the Max Planck Society, Berlin, using the double-corrected JEOL microscope JEM ARM200F (200 keV; equipped with image Cs-corrector, probe Cs-corrector, Gatan imaging filter (GIF) and HAADF detector).

### 2.2. Quantification of Precipitate Dimensions and Determination of Strain Field within and Around Precipitates Using GPA

To quantify the development of the precipitate size during the aging process, ${\left[110\right]}_{\mathrm{Al}}$ oriented STEM dark field images of areas with a foil thickness, *t*, of approximately 150–200 nm were recorded for different aging treatments. In this orientation, two variants of T_1_ and one variant of θ′ are edge on (i.e., parallel to the electron beam) [33]. Therefore, the length (diameter) and width (thickness) of the plate shaped precipitates can be measured and used as an indicator of the growth process.

When quantitatively comparing precipitates of different aging conditions, it is necessary to take into account that the disk-shaped precipitates in the investigated volume are for the most part truncated due to the thinness of the TEM foil. Therefore, line lengths of precipitates in ${\left[110\right]}_{\mathrm{Al}}$ oriented images correspond to the true precipitate diameter only if the center of the precipitate is within the TEM foil. Otherwise, the line length represents the length of a chord. To allow a direct comparison between the different samples and aging conditions, it is necessary that the TEM foil thickness is approximately the same. For quantification of line lengths and number density as a function of time, ${\left[110\right]}_{\mathrm{Al}}$ oriented HAADF-STEM overview images of regions with a thickness between 150 nm and 200 nm were analyzed. Due to the chosen ${\left[110\right]}_{\mathrm{Al}}$ orientation, only those precipitates that are lying on the ${\left(001\right)}_{\mathrm{Al}}$, ${\left(\bar{1}11\right)}_{\mathrm{Al}}$ and ${\left(1\bar{1}1\right)}_{\mathrm{Al}}$ planes were imaged. Thus, precipitates that are not parallel to the incoming electron beam are not considered in the quantitative analysis. Therefore, only one third of the θ′-precipitates and only half of the T_1_-precipitates are imaged in ${\left[110\right]}_{\mathrm{Al}}$ orientation [33]. This needs to be corrected on calculating the number densities and volume fractions of the precipitates by multiplying by a factor of 3 (for θ′) and 2 (for T_1_), respectively. 

It was not possible to determine the length of the precipitates based on their gray scale contrast to the matrix using an automatic software-based method, since gray scales varied locally within each image. Therefore, the precipitates were traced manually on three separate image planes (using the software Photoshop), i.e., each family of precipitate orientation on one plane (see Figure 13 in [37]). This results in three images with discrete gray scale values which can be analyzed by image analysis software Esprit (version 1.9, Bruker Nano GmbH, Berlin, Germany). Besides line length, the x and y coordinates of the center of gravity of the line were also determined for each precipitate. 

The thickness of each x, y position was determined by EELS (see Section 2.1). The thickness of individual precipitates was determined from high-resolution HAADF-STEM images (see Section 2.1) and a mean value was calculated for the volume fraction calculation. For each condition, about 300 to 400 T_1_ and about 250 θ′ precipitates were considered.

A procedure was developed by Häusler [37] to determine the volume fraction of oriented disk shaped precipitates from 2D projections of a 3D volume as recorded in the TEM. Detailed derivation and validation are given there and are not repeated here. The volume fraction of the counted precipitates was calculated using the following equation [37]:

(1) $$\begin{array}{c} f_{\nu }\left(s_{i},c_{i},t\right)\\ =100\%\sum_{i=1}^{N}\frac{1}{2}[\left\{\frac{\left(\frac{t}{0.5665\cdot s_{i}+t}\right)\left(\pi {\left(\frac{s_{i}}{2}\right)}^{2}c_{i}\right)+\left(\frac{0.5665\cdot s_{i}}{0.5665\cdot s_{i}+t}\right)\left(0.8276\cdot s_{i}^{2}c_{i}\right)}{area\cdot \left(0.5665\cdot s_{i}+t\right)}\right\}\\ +\left\{\frac{\left(\frac{t}{1.133\cdot s_{i}+t}\right)\left(\pi {\left(\frac{s_{i}}{2}\right)}^{2}c_{i}\right)+\left(\frac{1.133\cdot s_{i}}{1.133\cdot s_{i}+t}\right)\left(0.8276\cdot s_{i}^{2}c_{i}\right)}{area\cdot \left(1.133\cdot s_{i}+t\right)}\right\}] \end{array}$$

with:

*s_i_* length of the line *i*
*c_i_* thickness of the line *i*
*t_i_* TEM foil thickness in the center of the line *i*
*area* size of the investigated area (i.e., the area of the TEM image)

Since the determination of *c_i_* for each precipitate is not possible, an average $\bar{c}$ was calculated. Up to 10 precipitates of each precipitate type and each aging treatment were recorded using HR-STEM images and the corresponding arithmetic mean value $\bar{c}$ was calculated.

Finally, the volume fraction, $f_{v}\;$, combined with the thickness and the diameter of the precipitates allowed calculating the number density, *N*, of the precipitates per unit volume using the following equation [19]:

(2) $$N=\frac{4\cdot f_{v}}{\pi \cdot \bar{c}\cdot {\bar{s}}^{2}}$$

with $\bar{c}$ being the mean thickness of precipitates and $\bar{s}$ the mean line length of precipitates. 

**Figure 2:**
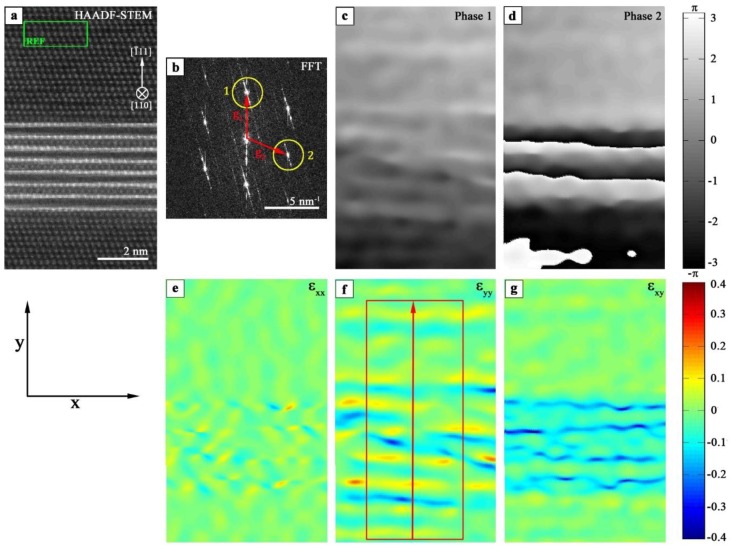
GPA procedure applied to a T_1_ precipitate (Type 4): (**a**) high-resolution HAADF-STEM image with reference area (REF) marked in green; (**b**) Fourier transformed HAADF-image with marked reflections 1 and 2 ($g_{1}=\;\bar{1}11$ and $g_{2}=00\bar{2}$ ); (**c**,**d**) calculated two-dimensional phase images 1 and 2 using reflections 1 and 2 in (**b**); and (**e**–**g**) two-dimensional strain maps *ε_xx_*, *ε_yy_* and *ε_xy_*. Scale bar in (c) and (d) is the same as in (a).

To obtain the strain fields within and around T_1_ precipitates, high-resolution HAADF-STEM images in ${\left[110\right]}_{\mathrm{Al}}$ orientation were evaluated by means of geometric phase analysis (GPA) [38] using the software ImageEval (University Bremen, Germany [39]). The GPA procedure is described in Figure 2. Before starting the strain analysis, the HAADF-STEM image was rotated to orient the precipitate in a horizontal position (Figure 2a). Then, an image area far from the precipitate with a defect free and unstrained structure was selected. This region was the reference area required for the analysis, and is abbreviated in the following with REF (see REF area in green in Figure 2a). Thereafter, the image was Fourier transformed, and subsequently the two linearly independent aluminum reflections $g_{1}=\;\bar{1}11$ and $g_{2}=00\bar{2}\;$were selected by a mask in the FFT image (Figure 2b; mask parameters: cosine damping and width of damping function in “units of mask radius” is 0.1). The two-dimensional phase images 1 and 2 were calculated (Figure 2c,d) based on the pixels within the mask area around the corresponding reflections 1 and 2. Finally, the two dimensional strain maps *ε_xx_*, *ε_yy_* and *ε_xy_* (see Figure 2e–g) were calculated from phase images 1 and 2.

**Figure 3:**
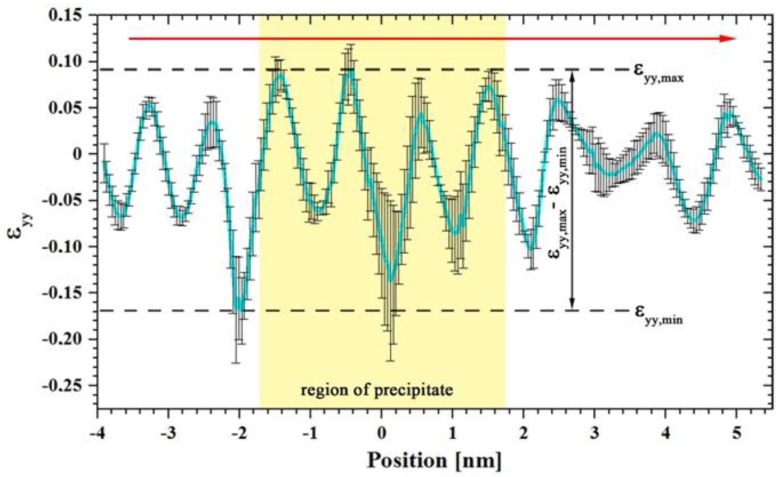
Line profile across the *ε_yy_* strain map shown in Figure 2f.

The strain fields within and around the precipitate correspond to the structural mismatch between fcc Al matrix and complex T_1_ precipitate. For better comparison of individual strain fields perpendicular to the precipitate–matrix interface, a line profile including error bars was generated across the two-dimensional *ε_yy_* strain field within the rectangular region marked in red. For the line profile, the strain values inside the rectangle were averaged along the x-direction and the corresponding standard deviations were calculated. The extreme values *ε_yy,min_* and *ε_yy,max_* were extracted from the one-dimensional *ε_yy_* strain profile (see Figure 3) and used to calculate the amplitude, i.e., *ε_yy,max_*–*ε_yy,min_*. This amplitude value reflects the distortion and, therefore the strain between the lattice planes in y-direction with respect to the reference area (fcc Al matrix). These values are used for the direct comparison of the strain in and around T_1_ precipitates of different types and thickness.

## 3. Results 

The microstructure of the initial state is characterized by elongated grains in L-direction (Figure 1) due to the extrusion process (not shown). The quantitative analysis of the grain sizes using the linear intercept method resulted in an average length of about 1111 μm and an average width of about 505 μm for the LT-ST plane (cf. Figure 1).

### 3.1. Creep Behavior

**Figure 4:**
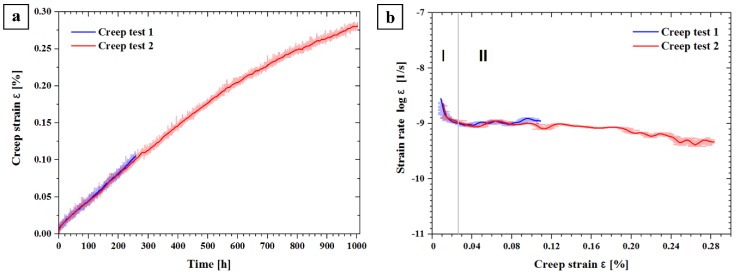
(**a**) Creep strain vs. time; and (**b**) strain rate vs. creep strain with creep stage I (primary) and II (secondary).

These tests primarily served to realize aging under an external load. Figure 4 shows the creep curves of the PA state obtained at a nominal stress of 155 MPa at 180 °C (*Creep test 1* and *2*). The two curves in Figure 4a almost coincide, which indicates the good reproducibility of the tests. *Creep test 1* was terminated after reaching 0.10% strain, and *Creep test 2* at 0.28% strain. From the creep strain evolution in Figure 4a, the creep rate, $\dot{\varepsilon }$, was calculated and plotted vs. creep strain, ε (Figure 4b). The figures show that both creep specimens were deformed to the secondary creep stage in which the creep rate is nearly constant.

### 3.2. Brinell Hardness

Brinell hardness measurements were carried out on the load-free aged samples and on the creep specimens to capture possible microstructural changes. Table 2 summarizes the results. During load free aging, the hardness decreases from 158 HBW in the PA state (*Ref. 0*) to 133 HBW after 257 h (*Ref. 1*) and 115 HBW after 1002 h (*Ref. 2*). Aging under applied stress (*Creep test 1* and *2*) results in similar hardness values as in the reference states.

### 3.3. Microstructure

#### 3.3.1. Peak Aged State

To interpret the hardness change during the aging and creep experiments, STEM investigations of the aged samples (without and with load) were carried out and compared to the PA state. 

**Figure 5:**
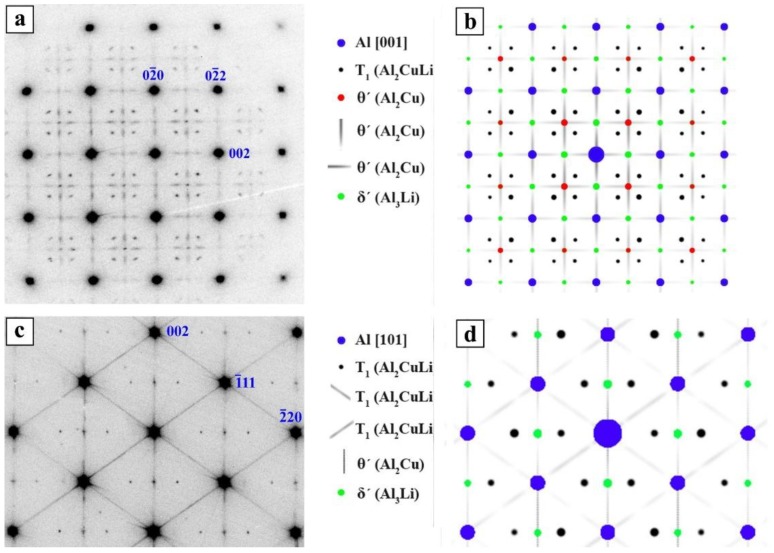
Energy-filtered experimental (**a**,**c**) and simulated (**b**,**d**) electron diffraction pattern of the PA state. Zone axis is ${\left[100\right]}_{\mathrm{Al}}$ in (**a**,**b**), and ${\left[110\right]}_{\mathrm{Al}}$ in (**c**,**d**) (adopted from [33]).

As previously reported [33], imaging the precipitate structure in TEM in ${\left[100\right]}_{\mathrm{Al}}$ and ${\left[110\right]}_{\mathrm{Al}}$ zone axis clearly show that only θ′ (Al_2_Cu) precipitates and T_1_ (Al_2_CuLi) precipitates are present in the PA state and no δ′ has formed. Experimental and simulated electron diffraction patterns of the PA-state are given in Figure 5. Slightly brighter dots at positions where reflections of the δ′ phase should appear are θ′ reflection rods lying perpendicular to the image plane. For a detailed interpretation of the diffraction patterns, see [33]. It is clear in Figure 5c,d that two variants of T_1_ and one variant of θ′ are edge on in the ${\left[110\right]}_{\mathrm{Al}}$ orientation.

**Figure 6:**
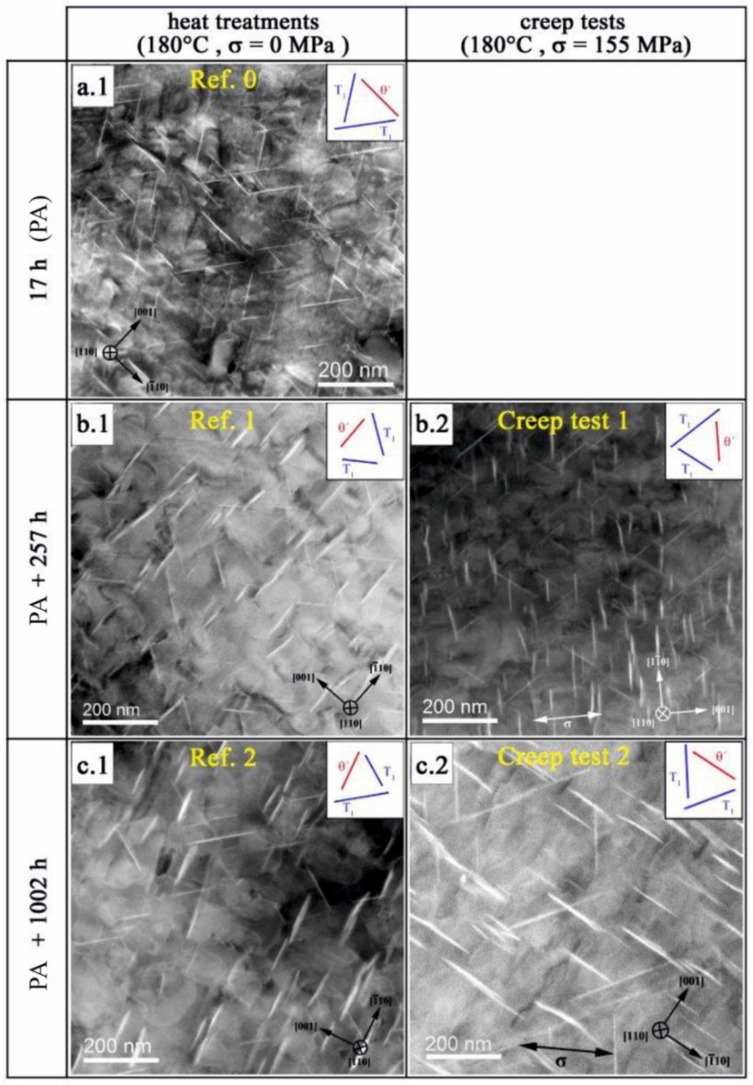
DF-STEM images of the microstructure as a function of aging treatment: (**a.1**) PA-state; (**b.1**,**c.1**) after aging without load; and (**b.2**,**c.2**) after loading under applied load (creep).

**Figure 7:**
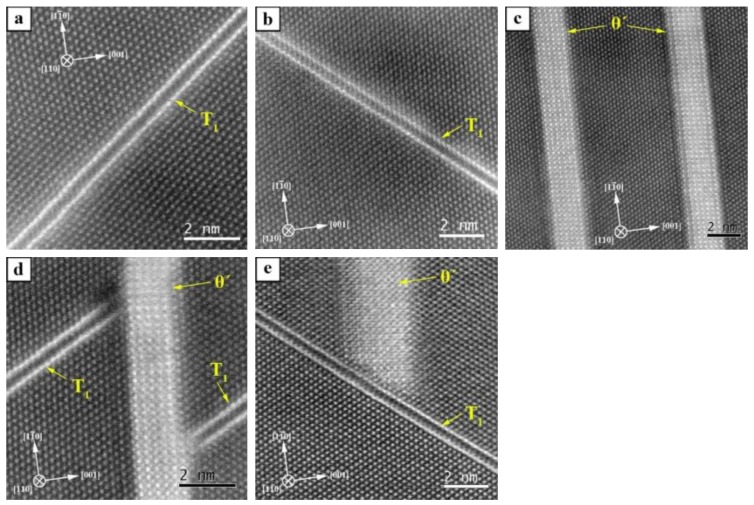
HR-STEM images of T_1_ and θ′ precipitates in the PA state: (**a**) T_1_ precipitate on ${\left(\bar{1}11\right)}_{\mathrm{Al}}$; (**b**) T_1_ precipitate on ${\left(1\bar{1}1\right)}_{\mathrm{Al}}$; (**c**) θ′ precipitates on (00$1{)}_{\mathrm{Al}}$; (**d**) θ′ precipitate stops the growth of two T_1_ precipitates; and (**e**) T_1_ precipitates terminates growth of a θ′ precipitate.

The micrograph of the PA state in Figure 6(a.1) shows a high number of plate shaped precipitates. Two variants of T_1_ (Al_2_CuLi) on the ${\left\{111\right\}}_{\mathrm{Al}}$ planes (marked in blue in the schematic in the upper right corner) and one variant of θ′ (Al_2_Cu) on the ${\left\{100\right\}}_{\mathrm{Al}}$ planes (marked in red) are visible. Based on high-resolution HAADF-STEM images (see Figure 7a,b), it is clear that, in this aging condition, all T_1_ plates consist of two bright layers separated by a relatively darker intermediate layer.

**Figure 8:**
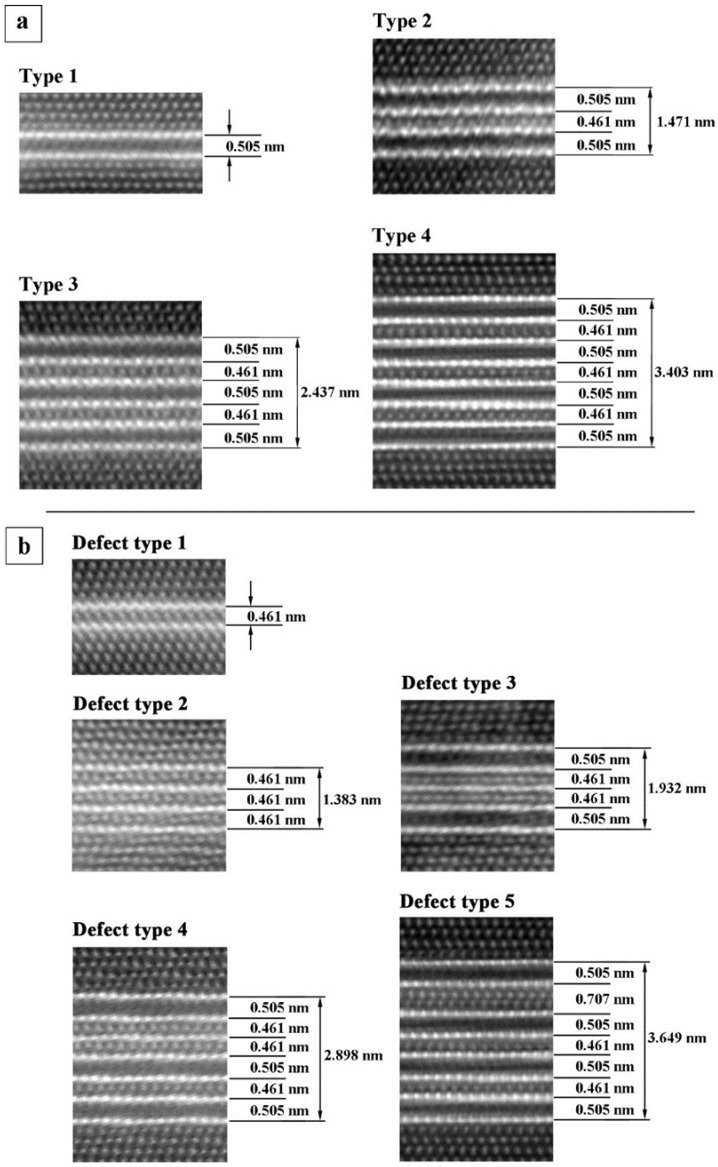
Types of T_1_ precipitates found in both the creep specimens and in the reference samples: (**a**) stacking sequences without defects; and (**b**) stacking sequences with defects.

The crystal structure of the single-layer T_1_ precipitates has been the subject of several studies [12,13,14,15,16,40]. There are, however, still slightly different ideas on the T_1_ structure. The similarity of all these structural models is the presence of a Li-rich layer surrounded by two Cu-rich layers. HAADF images of such an arrangement of atomic layers show two bright lines and a dark line in between [15,16] that were also observed in the present study. This stacking of T_1_ structure dominates in the PA state and in the following such stacking (two bright layers and one dark intermediate layer) is named “Type 1” or T-1 (see Figure 8a).

In addition to the T-1, several other stacking sequences of T_1_ precipitates were also found in the present study (see Figure 8). In the PA state, another T_1_ stacking was found having a bright intermediate layer with an intensity similar to that of the Al matrix. It seems that the Li atoms are missing or reduced in the intermediate layer of these precipitates and it can be assumed that the intermediate layer consists of Al atoms. This structure is called “Defect Type 1” or DT-1 (see Figure 8b). The T-1 and DT-1 T_1_ precipitates are 0.505 nm and 0.461 nm thick, respectively. This is in line with the fact that Li atoms are larger than Al atoms and hence their absence in the DT-1 precipitates results in a smaller thickness of the precipitate. In the PA state, 75% of T_1_ precipitates are of T-1 and 25% of DT-1 (cf. Table 3). Mean thicknesses are calculated and summarized in Table 4. Interestingly, T_1_ precipitates with mixed structure were observed as well. 

Figure 7b shows a T_1_ precipitate with mixed structure: the intermediate layer is rich in Al in the upper left region (bright intermediate layer) and rich in lithium in the lower right region (dark intermediate layer) of the image. These mixed typed precipitates were counted as both T-1 and DT-1. The two T-1 and DT-1 structures seem to be the elementary units for the T_1_ phase that are frequently observed during thickening, although it seems that the DT-1 stacking is not fully stable.

Comparing the dimensions of T_1_ and θ′ in the PA state (Figure 6a and Figure 7a, and Table 4), it is evident that the thickness of the θ′ precipitates is much larger than that of the T_1_ plates: it varies between 10 layers (1.56 nm) and 28 layers (4.4 nm) (Figure 7c). If a precipitate plate reaches another precipitate during growth, they do not penetrate each other; their length growth is rather stopped. Thus, the θ′ precipitate in Figure 7d terminates the growth of two T_1_ precipitates to its left and right side. 

Figure 7e shows another case where a θ′ precipitate meets a T_1_ precipitate and is stopped as well. A recent in-situ study by Liu et al. [41] showed the interaction of precipitates in close proximity: some precipitates completely vanished, while the growth of others was accelerated after coming in contact with each other.

#### 3.3.2. Effect of Further Aging

Further aging of the PA state at 180 °C for 257 h and 1002 h results in microstructural changes regarding mean size ($\bar{c}$ and $\bar{s}$), volume fraction, $f_{v}$, number density, *N*, and structure of the precipitates. Figure 6a.1–c.1 shows DF-STEM images of the microstructure of the PA state and the load free aged samples (*Ref. 1* and *Ref. 2*). It is apparent that the T_1_ and θ′ precipitates grew during 257 h aging. In this stage, it was found that all T_1_ precipitates thicken from a single layer to three layer with the same structure. The three-layer structure in this stage consists of a DT-1 layer sandwiched between two T-1 stackings. This kind of T_1_ precipitate is called “Type 2” (T-2). The T-2 precipitates have a thickness of 1.471 nm = 2 × 0.505 nm + 0.461 nm (see Table 4 and Figure 8a). The fact that all observed T_1_ precipitates after 257 h aging exhibit the same thickness and stacking (T-2) suggests that this structure was more stable than T-1 and DT-1 single layer precipitates. In fact, structurally, it is possible for either T-1 or DT-1 T_1_ precipitates to grow and form T-2 precipitate.

**Figure 9:**
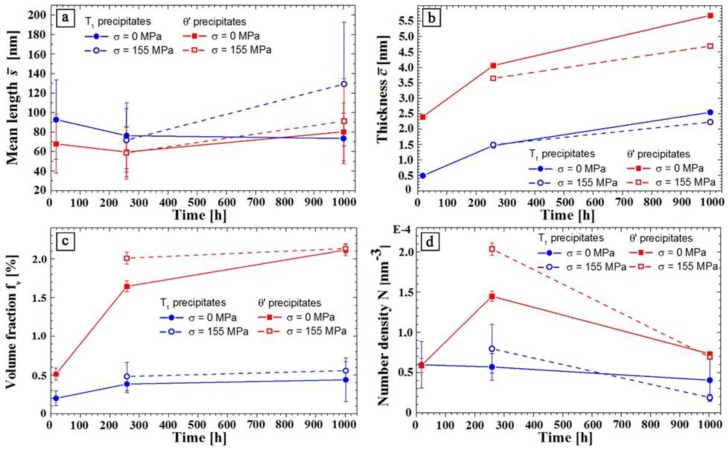
Evolution of the characteristics of T_1_ (blue) and θ′ (red) precipitates vs. aging/creep time: (**a**) mean line length $\bar{s}$; (**b**) mean thickness $\bar{c}$; (**c**) volume fraction $f_{v}$; and (**d**) number density *N*.

The line length of the T_1_ plates is approximately 76 nm, which is somewhat smaller than in the PA state (93 nm) but within the scatter of the length measurement (Table 4 and Figure 9a). Figure 9b shows that the θ′ phase (red symbols) behaves similarly, the thickness increases by a factor of 1.7 and the mean line length decreases somewhat but within the experimental scatter. The number density of θ′ precipitates increases by a factor of 2 as compared to the PA state, while that of the T_1_ precipitates (blue symbols) decreases slightly. Thus, it is not clear whether DT-1 precipitates disappear first and nucleate on the T-1 precipitates, or whether they can thicken on their own. It is to note that this picture of the thickening slightly deviates from the previous discussions on the structure of single-layer T_1_ precipitate, which assumes the interface Al layer would be part of the precipitate. 

Longer aging time of 1002 h leads to further thickening of θ′ precipitates up to 5.686 nm and a mean line length of about 80 nm. The mean thickness of the T_1_ precipitates increases to 2.546 nm but its length remains unchanged. In this case, several other types of T_1_ precipitate were observed as well. 

High-resolution STEM images of *Ref. 2* show that the stacking height and sequence of the T_1_ platelets increases markedly. Aging from 257 h to 1002 h, only 20% of T-2 stacking remain in the microstructure and the majority of the precipitates thicken in different stacking sequences (see Table 3 and Figure 8a): “Type 3” (T-3) and “Type 4” (T-4) stackings form, which consist of three and four T-1 stackings, respectively, separated by an Al-rich intermediate layer resembling the DT-1 structure. These are rather “regular” thickening of the T_1_ precipitate that are also observed in previous studies (for instance [10,19]). Furthermore, some T_1_ precipitates with irregular stacking frequency were found (see Table 3 and Figure 8b): Defect Type 4 (DT-4), which consists of a Type 4 stacking in which a Li-rich intermediate layer is absent; and Defect Type 5 (DT-5) that is a modified Type 4, in which one Al-rich layer is replaced by two Al-rich layers. Table 3 summarizes the frequency of the different stacking types for all investigated conditions. Closer inspection of the thickened structures suggests that all stacking sequences, except DT-5, which maintains a special structural defect, can be identified as multiple T-1 or DT-1 stackings with corresponding 0.505 nm and 0.461 nm thickness, respectively. 

After 1002 h, the number density of θ′ precipitates declines back to the value of the PA state, while that of T_1_ is unchanged. The volume fraction of the T_1_ precipitates increases only slightly (0.20% in the PA state, 0.38% after 257 h, and 0.44% after 1002 h) with time, while that of θ′ shows a drastic increase from 0.51% in the PA state to 1.65% after 257 h and 2.12% after 1002 h.

#### 3.3.3. Effect of Applied Load

The development of the microstructure under creep load is represented in Figure 6b.2,c.2 and the quantitative data are given in Figure 9.

Aging with applied stress results in only small length and thickness changes for both precipitates for 257 h compared to the load-free aging state *Ref. 1*. However, longer creep time (1002 h) results in growth in length direction for T_1_ while the thickness remains almost unchanged. The opposite is observed for θ′: the length changes only little, while the thickness is somewhat smaller than for stress free aging. The experimental data imply that the volume fraction and the number density of T_1_ phase seem not to be affected by an external stress. In contrast, creep loading for 257 h result in a 25% higher volume fraction and a 40% higher number density of θ′. It seems that the volume fractions for both precipitates and both loading conditions (load free/creep) reach a saturated value after 1002 h, which indicates that growth processes are completed, and coarsening will start on further aging.

The structure and stacking of the T_1_ precipitates, however, dramatically changes in the presence of external load. In contrast to the sample *Ref. 1* that contains only T_1_ plates of T-2, the loaded counterpart of this specimen (*Creep test 1*) contains T_1_ precipitates of T-1, T-3 and Defect Type 3 (DT-3) as well. In the DT-3 stacking, two DT-1 are sandwiched with two T-1 stacking sequences outside. This kind of defected structure was not observed during the load free aging even after 1002 h.

**Figure 10:**
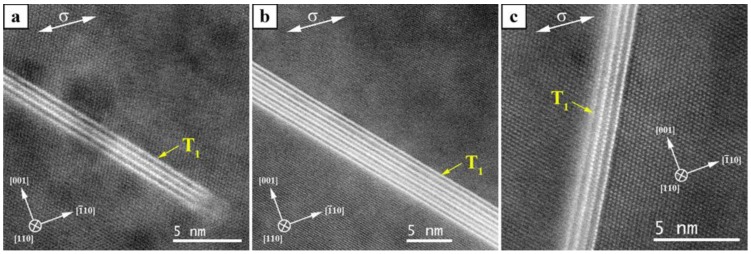
T_1_ plates of *Creep test 2* (155 MPa, 180 °C, 1002 h): (**a**) Type 2 on ${\left(\bar{1}11\right)}_{\mathrm{Al}};\;$(**b**) Type 4 on ${\left(\bar{1}11\right)}_{\mathrm{Al}}$; and (**c**) Type 2 on ${\left(1\bar{1}1\right)}_{\mathrm{Al}}$.

After 1002 h aging under load, Type 4 (T-4) precipitates also appeared and stacking T-3 disappeared. The T-3 and T-4 stacking can be considered as the next thickening sequence of a T_1_ precipitate with T-2 structure where further thickening of T_1_ precipitate occurs by sequential stacking of T-1 and DT-1. Figure 10 shows T_1_ precipitates after 1002 h of creep loading. The T_1_ precipitates in Figure 10a,b lie on the ${\left(\bar{1}11\right)}_{\mathrm{Al}}$ planes and have a stacking of T-2 and T-4, respectively. The T_1_ precipitate in Figure 10c lies on the ${\left(1\bar{1}1\right)}_{\mathrm{Al}}$ and is also of T-2. 

The angle between the external load direction σ and ${\left(\bar{1}11\right)}_{\mathrm{Al}}$ is about 46°, and that between σ and ${\left(1\bar{1}1\right)}_{\mathrm{Al}}$ is about 64°. Despite this relatively large angle difference of about 18°, which corresponds to a load difference of approximately 28 MPa perpendicular to the T_1_ precipitates interface, no differences regarding their size and thickness can be found between T_1_ plates on the ${\left(\bar{1}11\right)}_{\mathrm{Al}}$ and on ${\left(1\bar{1}1\right)}_{\mathrm{Al}}\;.$ The load difference was calculated by resolution of the external load *σ*:

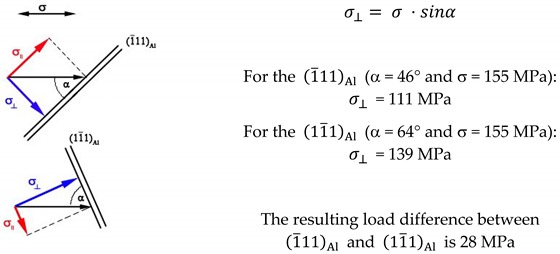
(3)

#### 3.3.4. Strain Measurements Using GPA

**Figure 11:**
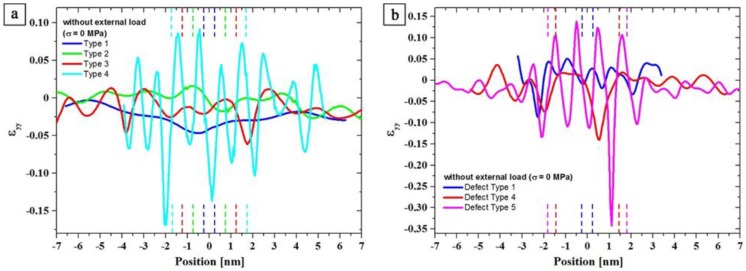
*ε_yy_* line profiles for different thickness and stacking of T_1_ precipitate types for load free aging: (**a**) defect free stacking (Types 1–4); and (**b**) T_1_ stacking with defects (Defect Types 1, 4, and 5). Position 0 refers to the mid thickness (*c*/2). Vertical dashed lines indicate location of matrix/precipitate interface.

To understand the thickening mechanism for T_1_ precipitates, different stackings and thicknesses of the precipitates were analyzed with respect to the corresponding strain states. This was done by employing GPA analysis, which provides information on the local displacement in and around the precipitates compared to the (stress free) reference Al matrix. In the following, the strain states for different stacking sequences are presented and used as an indication whether the lattice deformation in and around precipitates correlates to the stacking thickness or the stacking type. Figure 11 shows the comparison of *ε_yy_* line profiles of defect free T_1_-Types (Figure 11a) and T_1_-Defect Types (Figure 11b) of stress-free aged samples. The matrix/precipitate interfaces are indicated by dashed vertical lines. It is clear in Figure 11 that the variations occur inside and outside of the precipitates, i.e., lattice planes within the T_1_ plate as well as in the matrix are distorted in y-direction. It is evident that a significant increase in both the amplitude and the frequency of the variation of the *ε_yy_*-component occurs as a function of the stacking height (i.e., precipitate thickness).

In Figure 11a, the smallest strain amplitude of about 5% is obtained for the stacking of T-1 (blue curve) and T-2 (green). The amplitude in strain variation increases slightly for T-3 (red) compared to the previous stacking types. For T-4 (turquois), the strain variation is going up dramatically, up to five-fold the strain of T-1. Defect stacking DT-1 to DT-5 show similar characteristics (Figure 11b). The strain amplitude for DT-1 (blue) is about 6%, slightly more than the strain amplitude for the stacking T-1 and T-2. This means that thickening of DT-1 to DT-2 leads to a small reduction in strain energy. In fact, the T-2 stacking maintains the lowest strain among all different types of T_1_ precipitate. Since during the stress-free isothermal heat treatment no T_1_ precipitates with a stacking sequence of DT-2 and DT-3 were found, the next higher stacking is DT-4 (red). Its strain amplitude is about three times larger than the amplitude of DT-1. The highest strain amplitude for all T_1_ stacking types is caused by DT-5 (magenta) which maintains a completely different defected stacking, as shown in Figure 8b.

## 4. Discussion

The precipitate phases formed in ternary Al–Cu–Li alloys depend on the Cu/Li ratio. For Cu/Li > 4 (on wt. % basis), the precipitation sequence is: α(ss) → G.P. zones → θ″ → θ′ → θ, with α(ss) being the supersaturated solid solution [1]. For Cu/Li ratios between 2.5 and 4, the reaction changes to: α(ss) → G.P. zones → G.P. zones + δ′ → θ″ + θ′ + δ′ → δ′ + T_1_ → T_1_. There is also evidence that, in certain ranges of composition at a given aging temperature, T_1_ forms either in (T_1_ + δ′) or in (T_1_ + θ′) phase region. The Cu/Li ratio in the alloy investigated in this study is 4. In a previous study, we showed that, in our high-purity alloy, only θ′ and T_1_ are present in the PA state and no δ′ is formed [33]. In addition, it is reported that the Li-content has to exceed >1.4% to form δ′, which is not the case in our alloy [1]. The current study shows that longer heat treatments beyond the PA state (both without and with external load) do not affect the phase formation, i.e., no other phases beside θ′ and T_1_ are found. The volume fraction of θ′ in our high-purity alloy is found to be higher than that of T_1_, which is different from results obtained in technical alloys (e.g., Al-alloy 2195, which has similar Cu, Li and Mn content but higher impurity level). It has to be noted that the aging temperature used in the present study is about 30 °C higher than the commonly used temperature, which might affect the precipitation sequence (i.e., the aging temperature may exceed the δ′ solvus line).

Dorin et al. showed that, with an appropriate choice of alloy composition and thermomechanical treatment, the microstructure becomes dominated by T_1_ precipitates [19]. Alloying elements such as Mg, Ag and/or Zr are known to serve as nucleation sites for the formation of T_1_ resulting in T_1_ being the dominant phase and the other phases are often not discussed due to their small amount. In the present study, the alloy was free of impurity elements, which means that the precipitates can only nucleate on lattice defects such as dislocations. This can be one reason the θ′ phase occurred as the dominant phase. 

Dorin et al. investigated the growth behavior of the T_1_ precipitates as a function of time in a duplex aging treatment (at 155 °C and 190 °C) using SAXS measurements [42]. After 100 h, the system reached its maximum volume fraction. This agrees well with the observations of the present study, where the T_1_ volume fraction remains unchanged after 257 h or even earlier (see Figure 9c). The volume fraction of T_1_ in the crept specimens seems to be a little higher than in the load-free aged sample after 257 h and 1002 h. However, the difference is small and within the experimental error. 

Similar to previous work [15,42], we also observed only single-layer T_1_ precipitates after the PA treatment. The structures of the precipitates were, however, different (Table 3). In the present work, two types of single-layer T_1_ precipitates, T-1 and DT-1 on the {111} planes, were characterized in the PA state with thicknesses of 0.505 nm and 0.461 nm, respectively. While the structure of T-1 stacking confirms well the previous observations, the DT-1 stacking is not reported previously. For T-1 stacking, there are different structural models presented. In the Huang and Ardell [13] model, T-1 consists of an Al + Li layer surrounded by two Al + Cu layers and an Al + Li layer between the Al + Cu layer and the Al matrix. Howe et al. [12] supposed that T-1 is a pure Li layer surrounded by an Al + Cu layer. In both structural models, the Cu and Li atoms are disorderly distributed on the layers. The structural model of van Smaalen et al. [14] also assumes that a pure Li layer is surrounded by two Al + Cu layers. The difference to the model of Howe et al. is the displacement of Al atoms from the Al + Cu layer (with disordered Cu distribution) towards the central Li layer, i.e., a corrugated arrangement of the atoms. Furthermore, van Smaalen et al. assumed an Al + Li layer as a boundary layer to the Al matrix. Donnadieu et al. [15] analyzed HAADF images and SAXS results for all these models and found that the structure of the T-1 T_1_ precipitate is well described by van Smaalen model [14]. The results however struggled with the composition and atomic arrangement at the precipitate/matrix interface.

A follow-up experimental and DFT study by Dwyer et al. [16] presents a different sequence in T_1_ precipitates and resolved the discrepancy with respect to the composition of the interface layer. The Li-rich interfacial layer can only be imaged in <112>_Al_ orientation and is not observed in previous studies, as most of them focus on imaging in $<110{>}_{\mathrm{Al}}$ orientation. Furthermore, an extra layer of Al is observed close to the central Li layer in the T_1_ precipitates that is necessary for obtaining thermodynamic stability of this phase. The HR-STEM images of the T_1_ precipitates shown in the present work show no difference in intensity between the atomic columns of the Al matrix and that of the interfacial region of the T_1_ precipitates. However, the T_1_ precipitates were all imaged in $<110{>}_{\mathrm{Al}}$ orientation, in which the Li-sites in the interfacial layer are masked according to Dwyer et al. [16]. Recently, Kim et al. [17] suggested a modified structure of the van Smaalen model based on DFT studies that has a lower formation energy compared to all previous studies but only calculated at 0 K. However, a lack of understanding persists regarding the deformation processes at the interface layer of the T_1_ precipitate, which transform it into a distorted plane in the Al {111} matrix. The T_1_ precipitate with T-1 stacking occupies a multiple of ${\left\{111\right\}}_{\mathrm{Al}}$ atomic planes with $d_{\left\{1\;1\;1\right\}}$ = 0.223 nm. Comparing this to the theoretical width obtained for the van Smaalen structure (c = 0.9327 nm) [17] and assuming a positive strain (considering five ${\left\{111\right\}}_{\mathrm{Al}}$ atomic layer) one obtains ε_yy_ = 0.19939 (normal to the precipitate plane), which is a relatively large value. On the other hand, assuming a negative strain (considering four ${\left\{111\right\}}_{\mathrm{Al}}$ atomic layers), the strain becomes negligible (ε_yy_ = −0.00075). These are of course approximations since the other components of the structural deformation are not considered. Here the Li interfacial layers, which are displaced out-of-plane and towards the center of the precipitate can compensate the transformation strain.

The second type of single-layer T_1_ precipitates found only in the PA state is the DT-1. It contains a bright intermediate layer and smaller thickness (0.461 nm versus 0.505 nm for T-1 stacking) that exhibits an Al-rich intermediate layer instead of Li or Al + Li. 

Figure 7b shows a mixture of Type 1 and Defect Type 1 which suggests that the intermediate layer is progressively filled with Li atoms. This indicates that DT-1 can be a precursor of T-1, because it is only observed in the PA state (25% Defect Type 1 and 75% Type 1). Possibly, the parallel Al + Cu layers form first and Li atoms diffuse between the two Al + Cu layer in a later stage replacing Al atoms. During the filling with Li atoms, the Al atoms of the intermediate layer must now move towards the Al + Cu layers. Two diffusion paths are possible for this process: (1) perpendicular to the T_1_ surface and thus through the Cu-rich layers; or (2) parallel to the Cu-rich layers. Diffusion Path (2) implies that T_1_ plates with a large diameter grow slower in diameter than smaller ones: The lithium atoms have to reach the rim of the precipitate before they diffuse through them from the rim to the center of the precipitate. 

The observation of DT-1 type and analysis of thicker T_1_ precipitates suggests that the DT-1 stacking can be relatively stable, particularly next to the T-1 stacking. Although only 25% of T_1_ precipitates in PA state were found to be of DT-1 type, this stacking seems to be a necessary step for thickening T_1_ precipitate. Starting from a pre-existing DT-1, thickening can occur by the addition of a Cu layer (on the two sides of the precipitate) with an intermediate layer of Li atoms, i.e., a T-1 stacking. The resulting T_1_ precipitates have a T-2 stacking as shown in Figure 8a. Alternatively, a T-1 precipitate can thicken to a T-2 by addition of a DT-1 and a T-1 stacking, sharing their Cu layers (see Figure 8a). Extending the aging from PA state for an extra 257 h, a thickening reaction occurred such that:

2 (T-1) + (DT-1) → T-2(4)

The thickness of T-2 stacking is found to be 1.471 nm = 2 × 0.505 nm + 0.461 nm, which confirms the above thickening sequence (see Figure 8a and Figure 9b, and Table 4). In fact, T-1 and DT-1 stacking sequences are found to be elementary units of T_1_ precipitation and all subsequent thickening occur by multiplying the T-1 and DT-1 stacking. In this picture, the interfacial Al + Li layer in the single T_1_ precipitate, observed experimentally by Dwyer et al. [16], can rather belong to the Al matrix than the precipitate. This indeed explains the large distortion at the interface that was evidenced by Donnadieu et al. [15] as well as segregation of secondary alloying elements such as Ag at the interface [20]. 

**Figure 12:**
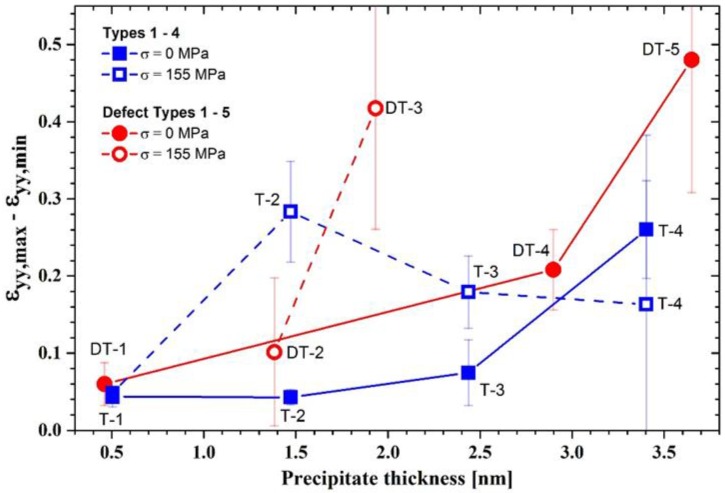
*ε_yy,max_* – *ε_yy,min_* for different T_1_ thickness and stacking after stress free aging and after creep testing calculated from the *ε_yy_* line profiles in Figure 11. Blue, Types 1–4; red, Defect Types 1–5. Error bar for T-2 is smaller than symbol height.

The GPA results show that thickening is accompanied by a change of the strain field in and around the precipitates. The difference of the strain values *ε_yy,max_* – *ε_yy,min_* was determined from the *ε_yy_* line profiles, as shown in Figure 11. These strain difference values of all stacking types of T_1_ are plotted as a function of precipitate thickness in Figure 12. One can see that strain difference value of the defect free stacking sequences after stress free aging (full blue squares) increases with precipitate thickness. For T-1 and T-2 stacking, the strain difference values are minimum. Indeed, the strain difference for T-2 stacking is slightly less than T-1 (4.3% and 4.4%, respectively) and DT-1 shows a somewhat higher strain difference value (6.0%). Hence, a reduction in the strain energy can be considered as one driving force for the thickening sequence [13]. 

A further thickening of T_1_ precipitate is accompanied by a large increase in the strain difference. The increase of the stacking height from T-2 stacking to T-3, for instance, increases the strain difference to 7.5%. Further growth in thickness results in a strain difference of 26% for T-4. In fact, formation of T-3 and T-4 stacking was only observed after very long aging or in the presence of external load (Table 3).

The application of an external load influences and disarranges the suggested sequence of thickening. The effect of external load enters evolution of precipitates in two ways [43]. If the system is heterogeneous, i.e., elastic constants of the precipitate and matrix phase differ, an external load *σ* generates an excess eigenstrain of $\sim \frac{\Delta \lambda }{\bar{\lambda }}\sigma$, where Δλ is the difference in elastic modulus and $\bar{\lambda }$ is the average elastic modulus of the mixture. The second effect is due to volumetric energy introduced by the external load. While the first contribution leads to morphological modification of dispersed precipitates, the second contribution results in a change in the fractions of the precipitate and matrix phases.

According to Figure 12, stacking of T-4 appears to be energetically more favorable than T-2 and T-3 stacking in creep tested samples (open blue squares). Moreover, it appears that the DT-2 is energetically more favorable than the DT-3 in the crept specimens (open red circles). For the interpretation of the strain amplitude values of the crept specimens (red and blue open symbols in Figure 12), it has to be noted that the strains were measured after unloading and the direction and the magnitude of the external load which acts on the precipitates during aging under creep, was not taken into account. Thus, the selection of the reference region (REF) does not represent a strain-free region during the aging under load and the *ε_yy_* measurements can only be taken as guide values in this case. To overcome this limitation, in-situ TEM studies have to be carried out, which were beyond the scope of the present study. 

Furthermore, attention should be paid to the role of dislocations in the growth and thickening of T_1_ precipitates that can facilitate nucleation of new layers on top of existing layers [10]. Dorin et al. [42] reported an abrupt acceleration in T_1_ thickening, when increasing annealing temperature from 155 to 190 °C, while the rate of lengthening (diameter) of the precipitate was not changing much. This observation suggests a thermally activated mechanism of thickening which could involve the nucleation of a new layer on the surface of the precipitate. This nucleation barrier can be greatly reduced when occurring at the intruding dislocations, as proposed by Cassada et al. [10,11]. Different studies have shown that the interaction among alloying elements, such as Mg, Cu and Zr, and dislocations can affect the nucleation of T_1_ phase [21,44,45]. A similar influence on the thickening mechanism of the precipitate may be assumed, possibly involving a modified nucleation process. In this scenario, the contribution of solute atoms to the thickening can be either due to a reduction in interface energy or elastic energy (in the vicinity of the precipitate), as discussed by Bourgeois et al. [46] for θ′ precipitation in a Sn-microalloyed Al-Cu system. Although secondary alloying elements are absent in the current alloy, it is likely that Li and/or Cu play a similar role, viz. reduces the interface energy, elastic energy or both, by segregating at the T_1_ interface or around it, which leads to the observed thickening sequence in the current study.

Comparing the strain difference values of the defect-free T_1_ structures (full blue squares) with those of the defected structures (full red symbols) after load-free aging, it is found that the defected structures (DTs) are associated with higher strain differences compared to the related defect free stacking (Figure 12). This interpretation is supported by the absence or small volume fraction of T_1_ precipitates with defected structure after aging without load (*Ref. 1* and *2*, cf. Table 3). The thickening sequence of the T_1_ precipitates in load-free isothermally treated samples is given by the increasing strain field/difference value as follows:

(T-1) + (DT-1) → (T-2) → (T-3) → (DT-4) → (T-4) → (DT-5)(5)

The analysis of strain differences suggests that the alternative sequence of the T-1 and DT-1 stacking, at least for T-2 and T-3 thickening can be driven by the reduction in the strain energy. In addition, the absence of DT-2 and DT-3 stacking can be explained by the formation of the energetically favored T-2 and T-3. Moreover, once the precipitates are of T-2 and T-3 stacking form, the stacking height for DT-2 and DT-3 has already been exceeded and the next stacking with defects is DT-4.

The current results show that the stacking sequence of the T_1_ precipitates changes depending on the aging condition. The precipitates thicken with ageing time. In the current study, no ledges have been found on the T_1_ precipitates while they were reported in early works of Cassada et al. [10,11]. The thickening of T_1_ precipitates were previously observed after a duplex aging treatment (at 155 °C and 190 °C) [42]. The single-layer precipitate growth requires diffusion to the tip of the plates, while the thickening process requires diffusion to the broad faces of the (semi-)coherent interface [42]. 

Growth by ledges is well known for precipitate plates in different alloy systems [47]. The new phase must nucleate within a solid crystalline matrix and the nucleus has to minimize the interfacial free energy and the volume strain energy to overcome the activation barrier. Ledges are small mobile steps with coherent faces. Atomic transfer occurs across the disordered steps, resulting in a lateral movement of the ledges. Nucleation of ledges usually occurs heterogeneously, and the ledges may show a variety of configurations. The nucleation of ledges is difficult (even in technical alloys) and it is assumed that they form by a heterogeneous nucleation process [48]. Bourgeois et al. [46] showed that trace additions of certain elements may effectively support the ledge nucleation process. However, in our high-purity alloy, such elements are not available. The thickening often takes place by alternative stacking of T-1 and DT-1 single-layer plates. It seems that these alternative stacking is favored due to a reduction in elastic energy. The analysis of the strain differences (Figure 11 and Figure 12) showed that a thickening to T-2 stacking results in a decrease of the strain amplitude around and within the precipitate while any further thickening leads to an increase. In the case of an external load, a higher number of stacks with defects was found in the crept specimens, which suggest that the accompanying lattice distortion energetically boosts the incorporation of defects into the stacking (Table 3).

The growth mechanisms appear to be different for the θ′ precipitates. It has to be noted that formation and growth of θ′ precipitates is characterized by a ledge mechanism [49], which allows the new phase to nucleate in the solid state phase transformation process. Dahmen and Westmacott [49] developed a model for θ′ predicting that ledges of certain step height will form to minimize the transformation shape changes and volume changes. 

**Figure 13:**
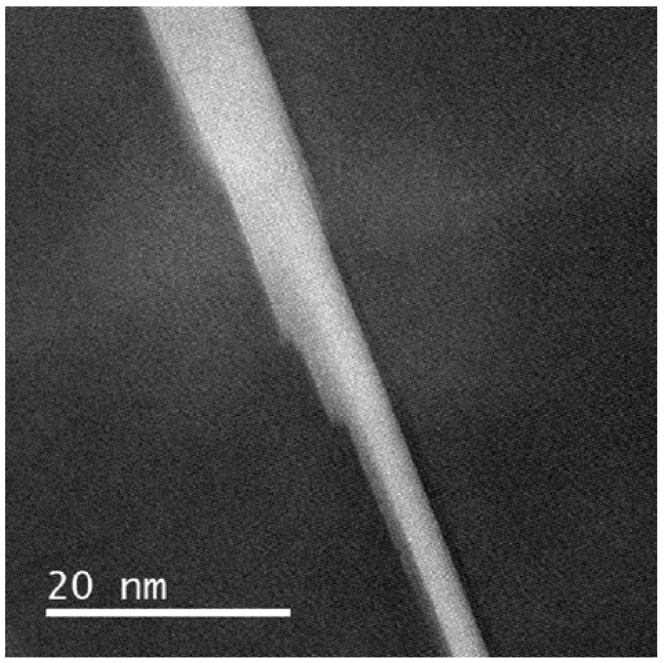
HAADF image of a θ′ precipitate documenting that the growth mechanism is dominated by ledges formed at the interface.

External load affects formation and growth of the θ′ precipitates. The experiments suggest that the length and arrangement of the precipitates is not much influenced by the external load (Figure 9a). This is due to the limited level of stress applied in this study. The volume fraction of the precipitates slightly increases under the external load (Figure 9c). The growth direction is roughly from the center to the rim of the precipitates (see Figure 13).

## 5. Conclusions

In the present study, the evolution of T_1_ and θ′ precipitates was studied and quantified during aging at 180 °C for 257 h and 1002 h under load free and under creep conditions. The results were obtained using TEM, HR-STEM and GPA strain measurements and discussed in the light of previous studies. In particular, the structure and thickening sequence of T_1_ precipitates were studied. The important findings are:

- Two elementary structures of single-layer T_1_ precipitate were characterized, one with a Li-rich (T-1) and another with an Al-rich (DT-1) central layer. The T-1 structure is very similar to the structure proposed in the previous studies with a thickness of 0.505 nm. The DT-1 structure, however, is observed here for the first time and it has a thickness of 0.461 nm. Single-layer T_1_ precipitates with mixture T-1 + DT-1 properties were observed as well that suggest DT-1 is a precursor for T-1 T_1_ precipitate.
- It was found that the thickening of T_1_ phase occurs by alternative stacking of the T-1 and DT-1 elementary structures. Based on the strain difference GPA measurements, a sandwich, T-2 = 2 (T-1) + (DT-1), T_1_ precipitate is the most stable thickened precipitate. All precipitates after 257 h aging are found to be in this form.
- Upon longer aging, further thickening of T_1_ precipitates was observed. Defected structures deviating from the alternative T-1 + DT-1 stacking sequence were observed as well, several of which were identified and studied in detail. In the absence of an external load, all thicker precipitates (including defected structures) carry a higher strain difference than T-2 stacking. In the presence of an external load, however, some of the defected structures form with lower strain difference that indicates they might have been preferred under loading condition. Furthermore, the frequency of precipitates with defected structures were found to be higher under loading condition.

While thickening occurs during aging, the length (diameter) of the T_1_ and θ′ precipitates were almost unchanged as compared to the PA state. Under external loading, the average length of T_1_ precipitates increases but it remains almost unchanged for θ′ precipitates. The volume fraction and the number density of T_1_ seem not to be affected by an external stress. In contrast, creep loading for 257 h result in a 25% higher volume fraction and a 40% higher number density of θ′. The volume fractions for both precipitates saturate after 1002 h, which indicates that growth processes are completed.

## Figures and Tables

**Table 1:** Sample designation and investigated material states.

Sample	Pre-Treatment	External Load (MPa)	Temperature (°C)	Time (h)
*Ref. 0*	PA (17 h/180 °C)	-	-	-
*Ref. 1*	PA (17 h/180 °C)	-	180	257
*Ref. 2*	PA (17 h/180 °C)	-	180	1002
*Creep test 1*	PA (17 h/180 °C)	155	180	257
*Creep test 2*	PA (17 h/180 °C)	155	180	1002

**Table 2:** Brinell hardness after aging without and with stress.

Sample	Aging/Creep Time (h)	Brinell Hardness HBW 2.5/62.5	
		load free aging	creep
*Ref. 0*	PA	158 ± 7	-
*Ref. 1/Creep test 1*	PA + 257	133 ± 2	132 ± 2
*Ref. 2/Creep test 2*	PA + 1002	115 ± 3	116 ± 2

**Table 3:** Relative frequency of T_1_ precipitate types after different aging treatments (see Figure 8 for details of the T_1_ types and defect types).

Specimen	T_1_ Type				Defect Type					Total Defects
	T-1	T-2	T-3	T-4	DT-1	DT-2	DT-3	DT-4	DT-5	
*Ref. 0*	75%	-	-	-	25%	-	-	-	-	25%
*Ref. 1*	-	100%	-	-	-	-	-	-	-	-
*Ref. 2*	-	20%	47%	20%	-	-	-	6.5%	6.5%	13%
*Creep test 1*	21%	52.7%	21%	-	-	-	5.3%	-	-	5.3%
*Creep test 2*	-	45.4%	-	27.3%	-	9.1%	9.1%	-	9.1%	27.3%

**Table 4:** Mean line length, $\bar{s}$ , with standard deviation and mean thickness, $\bar{c}$, of T_1_ and θ′ precipitates depending on the aging treatment.

Specimen	$\bar{s}\;$ (nm)		$\bar{c}$ (nm)	
	T_1_	θ′	T_1_	θ′
*Ref. 0*	93 ± 41	68 ± 30	0.494	2.402
*Ref. 1*	76 ± 34	60 ± 25	1.471	4.062
*Ref. 2*	74 ± 26	80 ± 30	2.546	5.686
*Creep test 1*	72 ± 32	59 ± 27	1.495	3.648
*Creep test 2*	129 ± 64	91 ± 44	2.231	4.694
